# Precise Identification of Higher-Order Repeats (HORs) in T2T-CHM13 Assembly of Human Chromosome 21—Novel 52mer HOR and Failures of Hg38 Assembly

**DOI:** 10.3390/genes16080885

**Published:** 2025-07-27

**Authors:** Matko Glunčić, Ines Vlahović, Marija Rosandić, Vladimir Paar

**Affiliations:** 1 Faculty of Science, University of Zagreb, 10000 Zagreb, Croatia; vpaar@hazu.hr; 2 Department of Interdisciplinary Sciences, Algebra University College, 10000 Zagreb, Croatia; ines.vlahovic@algebra.hr; 3 Department of Internal Medicine, University Hospital Centre Zagreb, 10000 Zagreb, Croatia; rosandic@hazu.hr; 4 Croatian Academy of Sciences and Arts, 10000 Zagreb, Croatia

**Keywords:** T2T-CHM13 assembly, alpha satellites, Cascading higher-order repeats, HORs, human chromosome 21 centromere, GRMhor algorithm

## Abstract

Background: Centromeric alpha satellite DNA is organized into higher-order repeats (HORs), whose precise structure is often difficult to resolve in standard genome assemblies. The recent telomere-to-telomere (T2T) assembly of the human genome enables complete analysis of centromeric regions, including the full structure of HOR arrays. Methods: We applied the novel high-precision GRMhor algorithm to the complete T2T-CHM13 assembly of human chromosome 21. GRMhor integrates global repeat map (GRM) and monomer distance (MD) diagrams to accurately identify, classify, and visualize HORs and their subfragments. Results: The analysis revealed a novel Cascading 11mer HOR array, in which each canonical HOR copy comprises 11 monomers belonging to 10 different monomer types. Subfragments with periodicities of 4, 7, 9, and 20 were identified within the array. A second, complex 23/25mer HOR array of mixed Willard’s/Cascading type was also detected. In contrast to the hg38 assembly, where a dominant 8mer and 33mer HOR were previously annotated, these structures were absent in the T2T-CHM13 assembly, highlighting the limitations of hg38. Notably, we discovered a novel 52mer HOR—the longest alpha satellite HOR unit reported in the human genome to date. Several subfragment repeats correspond to alphoid subfamilies previously identified using restriction enzyme digestion, but are here resolved with higher structural precision. Conclusions: Our findings demonstrate the power of GRMhor in resolving complex and previously undetected alpha satellite architectures, including the longest canonical HOR unit identified in the human genome. The precise delineation of superHORs, Cascading structures, and HOR subfragments provides unprecedented insight into the fine-scale organization of the centromeric region of chromosome 21. These results highlight both the inadequacy of earlier assemblies, such as hg38, and the critical importance of complete telomere-to-telomere assemblies for accurately characterizing centromeric DNA.

## 1. Introduction

Until recently, the centromeric region of the human genome has remained largely uncharted, limiting studies on the organization, variation, and function of centromeres. Recent technological advancements have facilitated the elucidation of the complete human chromosome assembly T2T-CHM13. This achievement has enabled the coverage of previously elusive complex structural variants in regions that were previously inaccessible [1,2,3,4,5,6,7]. These findings have significant implications for both health and disease. In a recent study by Altemose et al. [4], higher-order repeats were identified within the complete genomic sequences of the human centromeric region using a computational method similar to the previously introduced GRM method [8].

In the 1980s, it was discovered that human centromeres contain approximately 171 bp alpha satellite repeat monomers. These monomers are organized into tandem sequences of *n* monomers, known as *n*mer HORs [8,9,10,11,12,13,14,15,16,17,18,19,20,21]. The divergence between any two monomers within each HOR copy is significant, with variations ranging from ~20% to ~40%. However, these HOR copies are further organized in tandem, with the divergence between HOR copies typically being less than 5%. Monomers that exhibit less than 5% mutual divergence have been categorized as belonging to the same monomer type. Willard and colleagues observed that within each HOR copy, the constituent monomers mainly belong to distinct monomer types. This unique pattern, referred to as Willard’s-type HORs, has been extensively investigated using the limited sequencing data previously available, despite significant gaps in the centromeric region [22,23,24,25,26,27,28,29,30,31,32,33].

In *n*mer HOR arrays, the most common HOR copies, composed of *n* constituting monomers, are referred to as canonical. Copies within the same HOR array that contain inserts or deletions relative to the canonical HOR copy are referred to as variants. Identifying HORs within a given genomic sequence presents a highly intricate computational challenge, necessitating sensitive approximations. Until recently, this task was also hindered by significant limitations in sequencing technology. The GRM algorithm is a unique algorithm for precise identification of detailed HOR copies, both canonical and its variants, specifically for Willard’s-type HORs that do not repeat monomer types within HOR copies [8,34,35,36].

There are various algorithms available for identifying higher-order periodicities within a given genomic sequence (for example, [37,38,39,40,41,42,43,44,45]) due to the computational complexity of the problem. Notably, the GRM algorithm offers a distinct advantage in enabling the precise determination of HORs, facilitating the comprehensive identification of both the length and structure of all HOR copies. This was recognized in Ref. [4], which utilized the NTRprism algorithm. The authors of Ref. [4] noted that NTRprism is similar to the GRM method described in Ref. [46]. However, a limitation of this approach is its design specificity for Willard’s-type HORs, which are characterized by only one monomer of each type in each canonical copy.

To address this limitation, we have developed a novel algorithm named GRMhor, which is an enhanced iteration of our previous global repeat map (GRM) algorithm [8,36,46]. GRMhor extends its characterization beyond Willard’s-type HORs, focusing additionally on HORs with repeated monomer types within a canonical HOR copy. We refer to these extended HORs as Cascading higher-order repeats (Cascading HORs).

It is important to note that providing a rigorous description of the structural organization of alpha satellite HORs is a complex challenge, and discrepancies may arise between the results obtained from different methodologies. One significant advantage of the GRM and GRMhor tools is their ability to achieve high precision in identifying HOR copies and elucidating their structure. GRMhor not only detects peaks corresponding to alpha satellite HORs but also identifies smaller peaks that represent dispersed repeats (subfragments) not arranged in tandem. By utilizing the GRMhor algorithm, we can verify whether these additional peaks correspond to repeats, thereby enhancing the accuracy of our analyses.

Alpha satellite HOR units on human chromosome 21 were identified using cloned HOR units as hybridization probes under high-stringency conditions. This included both Southern blot analysis and in situ hybridization on metaphase chromosome spreads [17,47,48,49,50,51,52,53]. The nucleotide sequence of a predominant 11mer HOR unit has been determined in chromosomes 13 and 21 with minor variations between these chromosomes [49,53]. Additionally, in Ref. [47], four other alpha satellite subfamilies were detected: 7mer, 22mer, 28mer, and 9mer, while a 4mer subfamily was identified in Ref. [51]. The study noted that certain chromosome-specific mutations, which are not observed in restriction enzyme studies, may be undetectable by restriction enzyme digestion. These mutations may require alternative methods, such as direct sequencing, for their detection [54].

An analysis of the hg38 assembly was conducted in the pericentromeric region of human chromosome 21 [55]. The detection of HORs from the genomic sequence was performed in Ref. [8] using the GRM algorithm and dot matrix plots applied to the hg38 reference sequence. The primary HOR array identified in hg38 was the 8mer HOR, comprising 832 HOR copies. Additionally, variant HOR copies were detected with the following frequencies: 11mer (363 copies), 20mer (19 and 16 copies), 23mer (23 copies), 22mer (6 copies), 33mer (4 copies), and 16mer (3 copies) [8]. These findings for hg38 significantly differ from HORs previously identified using restriction enzymes, even for the primary HOR (8mer in the hg38 assembly compared to the 11mer determined by restriction enzymes).

Here, we present a precise identification and analysis of alpha satellite HORs and their subfragments by applying the GRMhor algorithm to the complete T2T-CHM13 assembly of human chromosome 21. Previous studies have identified various HORs on chromosome 21 using different algorithms, but without thorough analysis and precise identification of HOR arrays. For instance, Ref. [4] reported 11mer, 23mer, 22mer, and 13mer HORs, while Ref. [44] identified 11mer and 9mer HORs. Our approach provides a detailed examination and accurate mapping of these HORs, enhancing our understanding of their structure and organization.

## 2. Results

### 2.1. GRM (Global Repeat Map) Diagram

**Figure 1:**
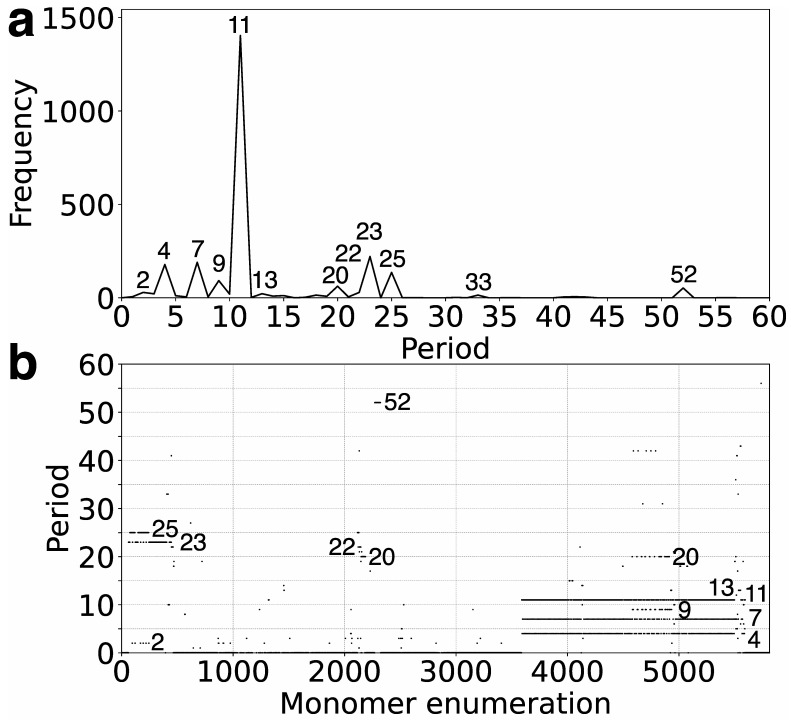
Global repeat map (GRM) diagram and monomer distance (MD) diagram for tandemly arranged alpha satellite monomers in the complete T2T-CHM13 assembly of human chromosome 21. (**a**) GRM diagram. Horizontal axis: GRM periods. Vertical axis: frequency of monomer repeat periods. The identified major GRM peak has a period of 11, while minor peaks occur at 4, 7, 9, 20, 23, 25, and 52. The significance of these GRM peaks (HORs or subfragment repeats) can be deduced from the MD diagram. (**b**) MD diagram. Horizontal axis: enumeration of tandemly organized alpha satellite monomers in the order of appearance in the GRM analysis of T2T assembly. Vertical axis: period—the distance between the start of one monomer and the start of the next monomer of the same type. The prominently distinct region featuring MD-line segments corresponds to 11mer HOR, while the additional MD-line segments occurring at periods 7 and 4 correspond to its subsegments. Short line segments with periods of 23 and 25 correspond to minor 23/25mer HOR, and the very short line segment with a period of 21 to 21mer HOR. A short line MD-line segment with a period comprising 52 MD points reveals the presence of two HOR copies with an exceptionally large period of 52. Furthermore, sporadic MD points are also observed.

In the first step, we identify tandemly organized alpha satellite monomers within the T2T-CHM13 assembly of human chromosome 21, enumerated in order of appearance in the genomic assembly. Employing the high-precision GRMhor algorithm, we compute the corresponding GRM diagram for this tandem array of monomers. Through this process, HORs and other monomer repetitions are discerned as peaks in the GRM diagram (Figure 1a). A distinct peak of period *n* (in units of 171 bp) represents an *n*mer HOR. The most prominent GRM peak observed in the T2T-CHM13 assembly of human chromosome 21 corresponds to the 11mer HOR.

The GRMhor algorithm, outlined in the Methods section, marks a significant advancement from the previous iteration of the global repeat map (GRM) algorithm. Originally designed to identify Willard’s-type HORs, characterized by the absence of repeating monomer types within a single HOR copy [1,2,3,4,5,6,7], the GRMhor algorithm surpasses this capability. It not only discerns Willard’s-type HORs but also extends its reach to identify HORs exhibiting multiple occurrences of the same monomer type within a single HOR copy, termed Cascading higher-order repeat (Cascading HOR) copies.

Moreover, the GRMhor algorithm facilitates the identification of various other types of monomer repeats, including intra- and inter-HOR-copy monomer repeats or tertiary HOR repeats, referred to as subfragments (SFs). In the T2T-CHM13 assembly of human chromosome 21, smaller GRM peaks occur at periods 4, 7, 9, 23, 25, and 52, with additional smaller peaks at 2, 13, 20, and 33 (Figure 1a). The nature of the periodic pattern corresponding to each GRM peak is determined by the MD diagram and the computation of repeat sequences.

### 2.2. MD (Monomer Distance) Diagram

The MD (monomer distance) diagram displays the relationship between the period of the monomer pattern and monomer enumeration (see Figure 1b). Each point on the diagram denotes a monomer enumeration on the horizontal axis and its corresponding distance to the next monomer of the same type in a sequentially organized monomer sequence. These points, referred to as MD points, in the case of HORs, are densely distributed, forming horizontal MD-line segments corresponding to a HOR, with the vertical coordinate presenting the period of the HOR. For a HOR, these MD points are densely distributed on the line segment, and with the naked eye, they resemble a continuous line in the interval corresponding to constituting monomers [7].

In the case of multiple parallel line segments, the top MD-line segment within a monomer enumeration interval corresponds to the *n*mer HOR array, where *n* indicates the period. As observed from the MD diagram (Figure 1b and Table 1), the most prominent MD-line segment corresponds to the major 11mer HOR. In the case of Cascading HORs, additional parallel MD-line segments may appear within the same monomer enumeration interval, displaying periods smaller than that of the 11mer HOR. These interspersed repeats, occurring within the HOR array—both the intra-HOR-copy and inter-HOR-copies—are termed subfragments [7]. As observed from the MD diagram (Figure 1b and Table 1), in the case of 11mer HOR, the GRM peaks of periods 7 and 4 correspond to subfragments.

**Figure 2:**
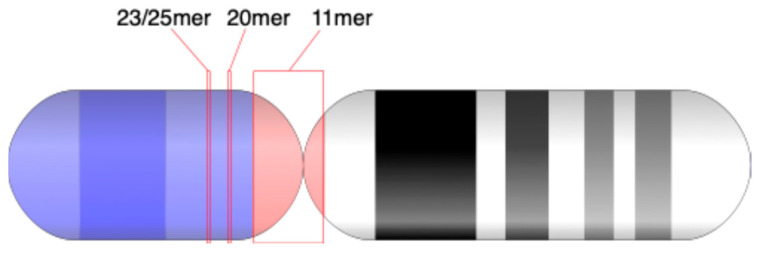
Ideogram of major alpha satellite HOR arrays within the centromeric region of the T2T-CHM13 assembly of human chromosome 21.

The shorter MD-line segments of different periods in the MD diagram correspond to other identified GRM peaks from Figure 1a: minor HORs (23/25mer HOR and 20mer HOR) and subfragments (periods 20, 9, etc.). The locations of 11mer, 23/25mer, 21mer, and 52mer HORs on chromosome 21 are depicted in an ideogram (Figure 2).

### 2.3. Aligned Scheme of Major Cascading 11mer HOR Array

**Figure 3:**
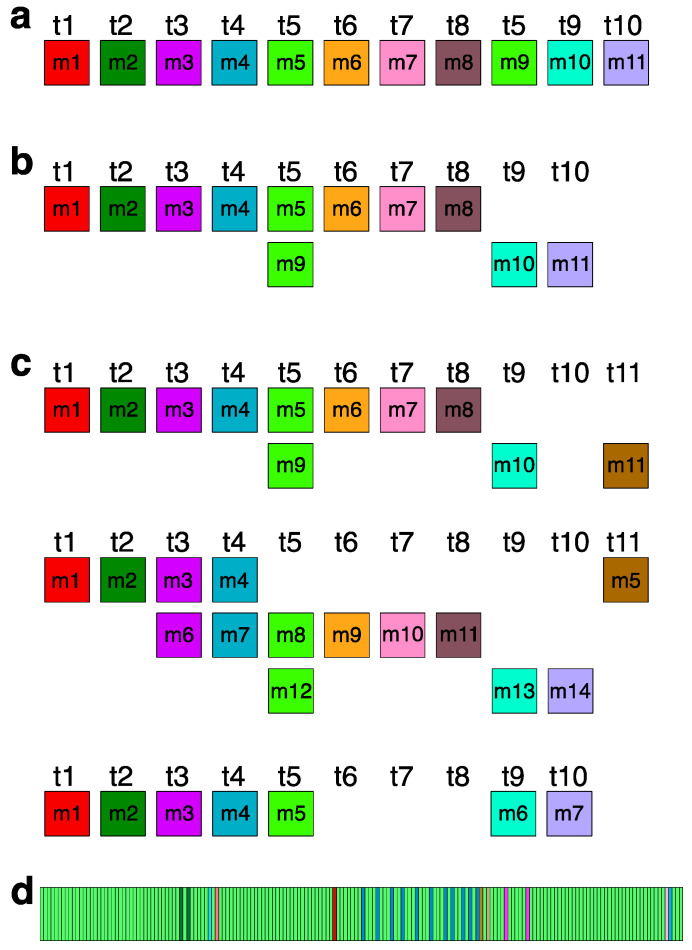
Aligned scheme of Cascading 11mer canonical HOR (**a**) The canonical 11mer HOR copy consists of *n* = 11 monomers designated as m1 to m11, belonging to 10 distinct monomer types *t*1 to *t*10, depicted in a linear monomeric scheme. The total number of monomer types in the canonical HOR copy is represented by τ = 10. Each monomer type is illustrated by a colored box, with different colors indicating different monomer types. (**b**) Cascading aligned scheme of the canonical 11mer HOR (*n* = 11, τ = 10), corresponding to the linear monomer scheme depicted in (**a**). Two monomers of the same type are aligned in the fifth column: monomer m5 of type t5 in the first row and monomer m9, also of type t5, in the second row. Monomer m10 of type t9 and monomer m11 of type t10 are in the second row. (**c**) Several examples of variant Cascading HOR copies corresponding to Cascading 11mer HOR (from Supplementary Figure S1). (**d**) Distribution of 11mer HORs within the HOR array. Each vertical rectangle represents a single HOR copy. Light-green rectangles indicate canonical 11mer HORs, while colored rectangles correspond to distinct variant types, defined based on their full HOR structure, as shown in Supplementary Figure S1. Colors are assigned as follows, in order of genomic appearance (left to right): dark green (11,036,265; 11,040,685), light blue (11,052,581), pink (11,056,320), dark red (11,118,525), blue (11,132,802; 11,139,938; 11,147,074; 11,152,340; 11,159,476; 11,166,614; 11,173,750; 11,177,148; 11,182,416; 11,185,814; 11,189,212), mustard (11,190,740), beige (11,194,820), pink (11,205,868; 11,216,408), pale mauve (11,288,641), and sky blue (11,291,875).

The graphical representations provided by the GRM and MD diagrams (Figure 1a,b) reveal the predominant arrangement of HORs within human chromosome 21, identified as the Cascading 11mer HOR. This array extends from 10,963,339 bp to 11,302,559 bp in the T2T-CHM13 assembly. The alignment pattern of the Cascading 11mer HOR array, computed using the GRMhor algorithm, is depicted in Supplementary Figure S1. The canonical 11mer HOR, the primary component of this array, is visually depicted through a linear arrangement of its constituent 11 monomers (Figure 3a) and redefined based on its monomer repetition in the Cascading HOR scheme (Figure 3b). Figure 3c showcases several examples of variant HOR copies corresponding to the Cascading 11mer HOR.

The total number of identified 11mer HOR copies is 182. Of these, 157 (86%) are canonical HOR copies. The consensus sequence of the 11mer canonical HOR copy is provided in Supplementary Table S1. While the HOR copies are arranged in tandem, directly adjacent to each other, the subfragments constitute their segments, being approximately equidistant but lacking direct mutual contacts. The graphical representation of the distribution of 11mer HOR canonical and variant copies within the HOR, spanning positions 10,963,339 to 11,302,559 on human chromosome 21, is illustrated in Figure 3d. From the central part of this distribution, it is evident that during the duplication of entire HORs, a mutation occurred within the canonical HOR, resulting in the transformation of the canonical HOR into a variant HOR. Subsequently, duplications proceeded with four, three, or two copies of HORs, resulting in the pattern CCCVCCCVCCVCCCVCCCVCCCVCCCVCVCCVCVCV, where ‘C’ denotes canonical, and ‘V’ denotes variant copies of the same type of HOR. Other variants of the HOR occur randomly within the HOR array.

### 2.4. Subfragments of Periods 4 and 7 in 11mer HOR Array

**Figure 4:**
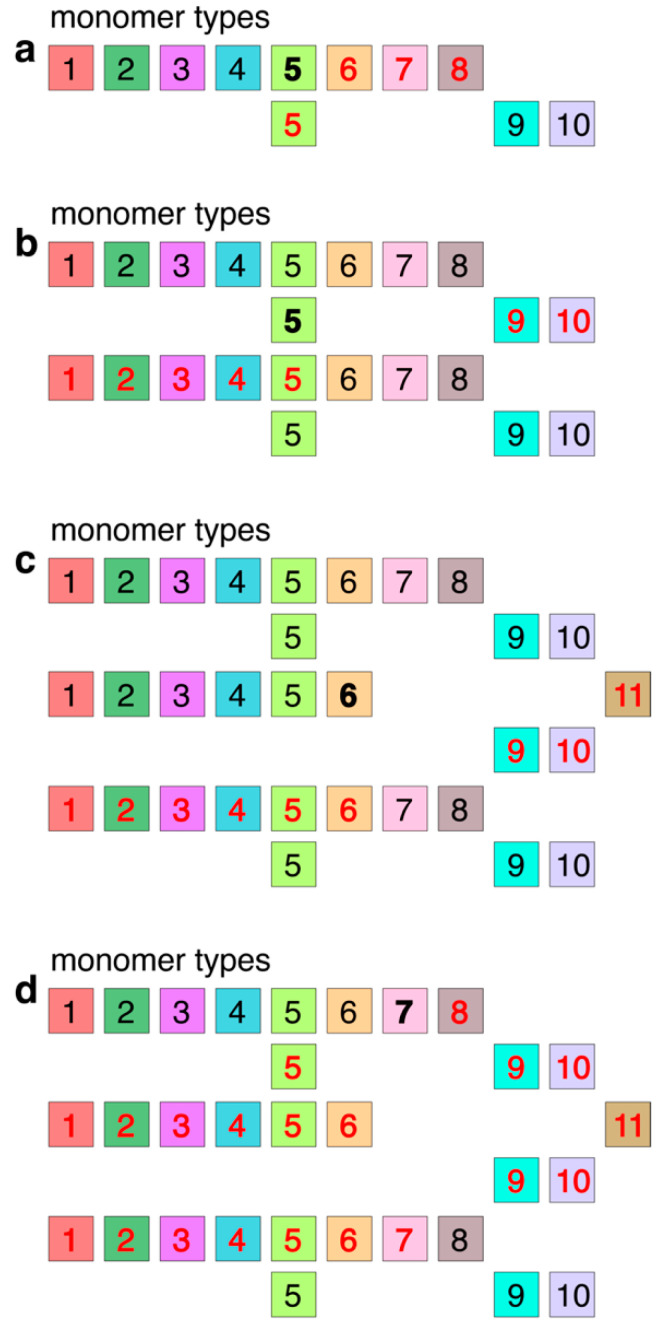
Origin of periods of subfragments from a graphical presentation of Cascading 11mer HOR copies. (**a**) Subfragments of period 4: 5→6→7→8→5. (**b**) Subfragments of period 7: 5→9→10→1→2→3→4→5. (**c**) Subfragments of period 9: 6→11→9→10→1→2→3→4→5→6. (**d**) Subfragments of period 20: 7→8→5→9→10→1→2→3→4→5→6→11→9→10→1→2→3→4→5→6→7.

Within the Cascading HOR presentation of the canonical 11mer HOR copy (Figure 4a), a periodicity of 4 also emerges, for example, going in four steps from the monomer of type 5 (bold) in the first row to the monomer of type 5 in the second row: 5→6→7→8→5.

Additionally, within a pair of canonical 11mer HOR copies in Cascading HOR presentation, a period of 7 also emerges, for instance, progressing through seven steps from the monomer of type 5 (bold) in the second row of the first HOR copy to the monomer of type 5 in the first row of the second HOR copy (Figure 4b): 5→9→10→1→2→3→4→5.

The period-4 and period-7 patterns observed in restriction enzyme studies of human chromosome 21 (Refs [47,51]) may correspond to these subfragments within the monomer encryption region of the 11mer HOR (MD-line segment of period 11 in Figure 3b).

### 2.5. Subfragments of Periods 9 and 20 in 11mer HOR Array

The most frequent variant among 11mer HOR arrays is a triplet configuration featuring Cascading 11mer HOR copies: the first and third HOR copies are canonical, while the middle copy exhibits modifications, notably with the deletion of the final two monomers in the first row (see Figure 4c). Within this triplet of HORs, a period of 9 emerges, going from one monomer to the next over nine steps, from monomer 6 (bold) in the second HOR copy of the triplet (Figure 4c): 6→11→9→10→1→2→3→4→5→6.

A period of 20 appears, for example, going from monomer to monomer in 20 steps from the monomer type 7 (bold) in the first row of the first HOR copy to the monomer 7 in the first row of the third HOR copy (Figure 4d):

7→8→5→9→10→1→2→3→4→5→6→11→9→10→1→2→3→4→5→6→7.

The period of 9 observed in restriction enzyme studies of human chromosome 21 (Ref. [47]) might correspond to the period-9 subfragment within the monomer encryption region adjacent to the 11mer HOR (short MD-line segment of period 11 in Figure 3b).

### 2.6. Aligned Scheme of 23/25mer HOR Array

**Figure 5:**
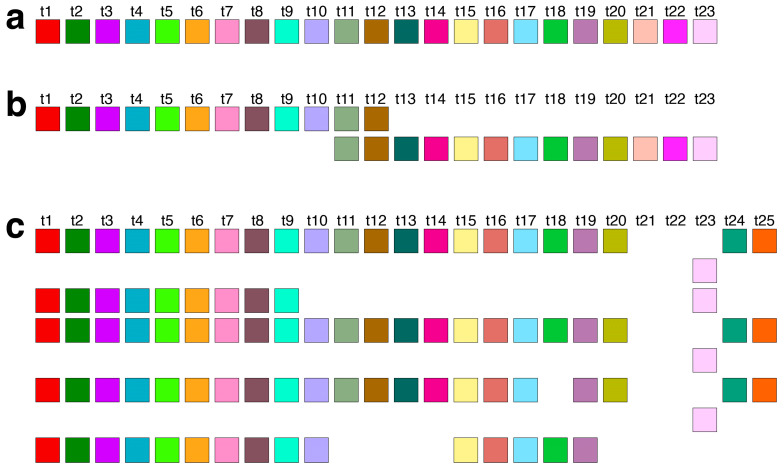
Graphical presentation for 23/25mer HOR array. (**a**) Graphical presentation of a Willard՚s-type canonical 23mer HOR copy. The constituent monomers are designated as t1, t2, t3, …, t23, each of a distinct type from the others. Various monomer types are represented by distinct colors. (**b**) Aligned graphical presentation of canonical 25mer HOR as a two-row Cascading HOR copy. The first row of the HOR copy contains monomer types t1-t12, while the second row contains t11-t23, with repeated monomers of types t11 and t12 aligned. (**c**) A segment comprising the five 23mer variants of 23/25 HOR copies.

The initial 14 HOR copies of the 23/25mer HOR array are canonical, arranged sequentially within the genomic interval spanning from 8,180,952 bp to 8,233,839 bp, as follows: 23mer, 2 × 25mer, 23mer, 4 × 25mer, 6 × 23mer (see Supplementary Figure S2). Each canonical 23mer HOR copy is of Willard’s type, comprising 23 different monomer types denoted as t1, t2, t3, …, t23 (Figure 5a). Conversely, each canonical Cascading 25mer HOR copy contains the same 23 monomer types as the 23mer HOR copies, with the exception of duplicated monomer types t11 and t12, which are aligned (Figure 5b).

This highly regular segment of 14 canonical 23/25mer HOR copies is succeeded by a cluster of 5 variant HOR copies (Supplementary Figure S2). While these variant copies maintain alignment with the monomer types of canonical HOR copies, specific monomer types are absent in the second, fourth, and fifth HOR copies (t10–t22, t18, and t11–t14 plus t20–t23, respectively) (Figure 5c). Furthermore, the t21 and t22 monomers are replaced by a new monomeric doublet t24–t25, in comparison to canonical HOR copies. The consensus sequence of the 23/25mer canonical HOR copy is provided in Supplementary Table S2.

### 2.7. Aligned Scheme of Diverged 21mer HOR Array

**Figure 6:**
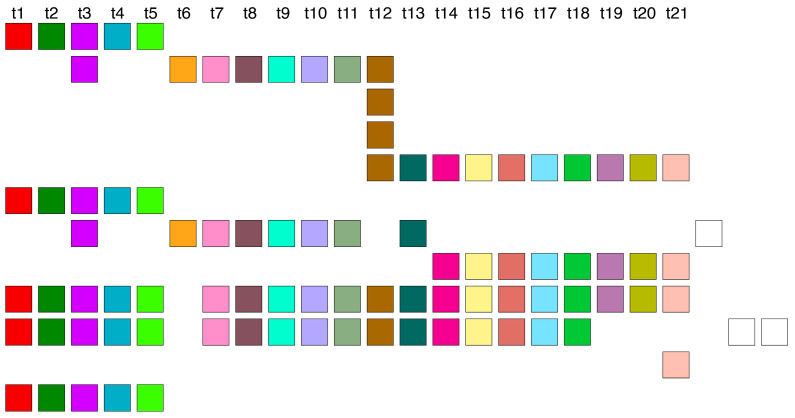
Aligned scheme of diverged 21mer HOR array. HOR copies are identified on the basis of the alignment of monomer types. This array consists of five HOR copies characterized by 21 aligned monomer types. This array is highly divergent, with all HOR copies being mutually different.

The third significantly divergent HOR array is composed of 21mers, each consisting of 21 distinct monomer types (Figure 6). All monomer types within this 21mer HOR are unique and differ from monomer types found in the 23/25mer HOR and 11mer HOR arrays. This HOR array is highly divergent, without the presence of a canonical HOR copy, and, therefore, the corresponding GRM peak cannot be observed. However, some of the corresponding rare MD points are visible at period 21.

### 2.8. 13. Mer Subsequence Duplication in Triplet of 23/25mer HOR Copies Generating a Monomer Subsequence Repeat Pattern of Period 33

**Figure 7:**
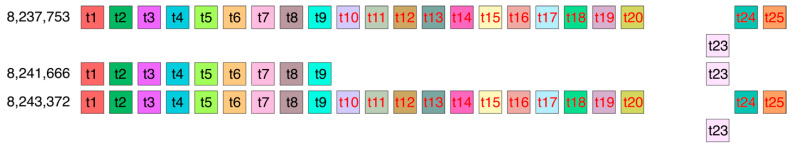
Triplet of variant 23/25mer HOR copies generating a pattern of period 33 among monomers. Three variant 23/25mer HOR copies from Figure 5 contain an identical 13-monomer subsequence for types of monomers in the first and third HOR copies (colored red). Distance from the start of t10 in the first HOR copy to the start of the next t10 in the second HOR copy = 13 + 1 + 9 + 1 + 9 = 33 monomer lengths. Analogously, the same distance of 33 is periodically repeated for t11 to t11, t12 to t12, …, t25 to t25. This periodicity gives rise to a weak GRM peak of period 33 and slightly visible points in an MD diagram.

Let us consider the distance d(t10) between the start of a monomer of type t10 in the 8,237,753 23/25mer HOR copy and a monomer of the same type t10 in the 8,243,372 23/25mer HOR copy (Figure 7). This distance is equal to the sum of the lengths of 13 monomers (monomer subsequence in the first row, colored red) and the length of 20 monomers between the end of the first 13 monomer subsequence (red) and the start of the second monomer subsequence (red) in the fourth row of Figure 7. In this way, d(t10) = 13 + 20 = 33 (in units of monomer length). Analogously, for the other 12 monomers in the subsequence of 13 monomers, there is d(t11) = d(t12) = … d(t20) = d(t24) = d(t25) = 33 (in units of monomer length). Thus, we obtain that the MD-point frequency of the corresponding period 33 is very small (13) in comparison to other frequencies in Table 1. Therefore, the corresponding GRM peak is very small, and the frequency points do not form a full MD line. Thus, there is no 33mer HOR in the T2T-CHM13 assembly of human chromosome 21, contrary to the hg38 assembly, which contains four 33mer HOR copies [35].

### 2.9. Absence of 33mer HOR and 8mer HOR in Complete T2T-CHM13 Assembly of Human Chromosome 21 Is Contrary to Results Obtained Using Hg38 Assembly

In the preceding HOR analysis of the hg38 assembly, a 33mer HOR array comprising four 33mer HOR copies was identified on human chromosome 21 (see Figure 3a in Ref. [35]). Conversely, in the complete T2T-CHM13 assembly of human chromosome 21, no 33mer HOR copies were detected. Instead, a minute elevation in the frequency of the GRM at period 33 is observed in the T2T-CHM13 assembly (Figure 1a); however, it does not align with the characteristics of a 33mer HOR. This underscores the deficiency of the hg38 assembly, specifically within the centromeric region.

Furthermore, the inadequacy of the hg38 assembly in the centromeric region becomes even more apparent in its failure to identify the primary alpha satellite HORs in human chromosome 21. The principal HOR array identified in the T2T-CHM13 assembly presented here is the 11mer, which contrasts with the previously identified 8mer in the hg38 assembly [35]. This finding aligns with recent results from Ref. [7] and early studies using restriction enzymes.

### 2.10. Novel 52mer HOR—The Longest Alpha Satellite Canonical HOR Copy Discovered in the Human Genome 

**Figure 8:**
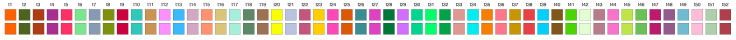
Aligned scheme of two 52mer HOR copies. The constituent monomers, designated as t1, t2, t3, …, t52, are distinct from one another. Different monomer types are indicated by unique colors.

In the GRM diagram for human chromosome 21, a prominent GRM peak emerges at period 52 (Figure 1a). Correspondingly, in the MD diagram (Figure 1b), a notable MD line comprising approximately 100 monomers appears at a monomer enumeration of ~2300, aligning with the aforementioned GRM peak. On the other hand, analysis utilizing the GRMhor algorithm reveals the presence of two complete 52mer HOR copies (Figure 8). The computed average divergence among monomers in each HOR copy is 17.7%, while divergence between the two HOR copies is much smaller, only 0.2%, consistent with the characteristic 52mer HOR pattern.

Moreover, examination of the MD-point frequency (Table 1) corroborates the existence of two intact 52mer HOR copies. Specifically, the computed number of corresponding MD points (52) precisely matches the count of monomers constituting the 52mer HOR copy (52). Notably, this 52mer HOR copy represents the longest alpha satellite HOR copy identified within the human genome to date.

### 2.11. Sequence Divergence Analysis of Consensus Alpha Satellite Monomers and HOR Copies

To support the classification of canonical and variant HORs, we performed a quantitative analysis of sequence divergence among consensus alpha satellite monomers and canonical HOR copies. Pairwise edit distances (expressed as percentages) were computed using the edlib algorithm and normalized relative to sequence length.

### 2.12. Intra-HOR Divergence

For each HOR type, we compared all alpha satellite monomer sequences within the same HOR unit, excluding self-alignments (i.e., comparisons of each monomer with itself). The goal was to assess the internal sequence heterogeneity of each HOR array. The results were as follows:

11mer HOR: mean = 22.30%, min = 5.26%, max = 31.58%.

23mer HOR: mean = 20.27%, min = 6.43%, max = 31.76%.

52mer HOR: mean = 17.68%, min = 8.19%, max = 28.40%.

These values confirm that all three HORs are composed of diverse alpha satellite monomers, with moderate to high sequence variation between constituent units. Notably, the 52mer HOR exhibits the lowest mean and maximum divergence, suggesting a slightly more homogeneous composition, which may reflect greater evolutionary stability or recent homogenization.

### 2.13. Inter-HOR Divergence

We also compared all monomers from each HOR to all monomers from the other HORs, with no exclusions. The aim was to quantify how distinct each HOR is from the others:

11mer vs. 23mer: mean = 26.21%, min = 17.44%, max = 38.95%.

11mer vs. 52mer: mean = 25.12%, min = 17.65%, max = 33.92%.

23mer vs. 52mer: mean = 20.12%, min = 8.82%, max = 33.76%.

The consistently high minimum divergence values between the 11mer and the other two HORs (~17%) indicate that they are composed of distinct sets of monomers. This supports their classification as independent canonical HOR families. Interestingly, the minimum divergence between 23mer and 52mer is significantly lower (8.82%), suggesting partial sequence overlap or a more recent common ancestry between these two arrays. This observation aligns with their physical proximity and architectural similarity, particularly in the context of cascading HOR structure.

### 2.14. Intra-HOR Divergence Among Canonical HOR Copies

To further examine the homogeneity of HOR arrays, we performed an additional analysis comparing the full sequences of canonical 11mer HOR copies—i.e., those composed of a complete and uninterrupted arrangement of 11 distinct or recurring monomers. The edit distance between every pair of canonical 11mer HOR copies was computed, yielding the following results:

Canonical 11mer HOR copies: mean = 0.90%, min = 0.00%, max = 2.57%.

These results reveal a remarkably high degree of sequence conservation among canonical 11mer HORs. The observed variation is minimal and consistent with expected low mutation rates in functionally constrained tandem repeats. Similar levels of homogeneity were also observed in the two 52mer HOR copies (see previous subsection), indicating that canonical HORs—once established—are maintained with high fidelity, likely due to concerted evolutionary processes such as unequal crossing-over and gene conversion.

These findings emphasize that the majority of sequence variation arises not from canonical HORs themselves, but from the presence of variant HOR copies embedded within the same arrays. These variants may result from localized mutations, insertions, or structural rearrangements and are clearly distinguishable from the core canonical units based on both alignment and GRMhor-derived MD profiles. The ability of GRMhor to resolve these subtle yet functionally relevant distinctions underscores its value for repeat architecture analysis.

Together, this divergence-based evidence supports the classification framework used in this study and demonstrates that both monomer-level and HOR-level sequence variation follow discernible patterns that correlate with HOR identity and structural integrity.

## 3. Discussion

The identification of the novel 52mer HOR—the longest canonical alpha satellite HOR unit in the human genome—alongside the previously unrecognized 11mer and 23/25mer HOR arrays in the T2T-CHM13 assembly, raises important questions regarding their potential functional significance. Alpha satellite HORs are central to the establishment and maintenance of centromere identity, in large part through their interaction with centromere-specific proteins such as CENP-A. The discovery of large, highly structured HOR units in chromosome 21 may point to a previously underappreciated structural basis for centromere function or chromatin architecture.

Furthermore, the presence of Cascading HOR types—where specific monomer types are repeated within a single HOR unit—suggests a higher degree of internal redundancy or modularity. This could enhance the robustness of centromere assembly, particularly in the face of mutation or chromosomal instability. The mixture of Willard’s and Cascading HOR architectures (e.g., in the 23/25mer region) may represent an evolutionary compromise between repeat uniformity and structural flexibility.

Finally, the comparative absence of such HOR structures in the hg38 assembly, and their detection only in the complete T2T-CHM13 assembly, underscores the importance of complete, high-resolution genome assemblies for the accurate characterization of centromeric elements. The findings presented here may contribute to future studies of kinetochore assembly, centromere evolution, and genome architecture in both normal and disease contexts.

Our findings build upon prior efforts to map alpha satellite sequences and centromeric structure in the human genome. In particular, Altemose et al. [4], using the HumAS-HMMER-HOR pipeline, identified several alpha satellite HOR arrays on chromosome 21, including 23mer, 22mer, 11mer, and 13mer units [4]. However, while these annotations captured general HOR presence, they did not resolve the full monomer-by-monomer structure, canonical ordering, or detect the presence of superHOR units, such as the 52mer HOR described here. Our approach provides detailed structural alignment, precise HOR boundary definitions, and identification of both canonical and variant forms. Notably, the 52mer HOR represents the longest alpha satellite HOR unit identified to date in the human genome; the previous longest-known HOR was the 34mer in the Y chromosome.

In addition to canonical HORs, our analysis uncovered numerous subfragment repeats, including 4mer, 7mer, 9mer, 13mer, 20mer, and 28mer units. These represent intra- and inter-HOR-copy substructures with defined monomeric composition and spacing. Interestingly, some of these *n*mer repeats were previously observed as subfamilies using classical restriction enzyme digestion methods [47,48,49,50,51,52,53] but without the structural resolution now enabled by GRMhor.

We also contrast our results with the hg38 assembly, where previous annotation identified an 8mer as the dominant HOR and a 33mer array with only four HOR copies [35]. These results differ substantially from our findings in the T2T-CHM13 assembly, further demonstrating the limitations of hg38 in centromeric regions and highlighting the importance of telomere-to-telomere assemblies for comprehensive repeat structure analysis.

Several limitations of our approach should also be acknowledged. First, GRMhor relies on consensus-based alignment of alpha satellite monomers, which may result in reduced sensitivity in highly diverged or mosaic regions. Second, while GRM and MD diagrams provide visually robust validation of repeat periodicity, they are not statistical models and do not currently offer confidence metrics. Third, our analysis focuses on a haploid assembly (CHM13), and further validation across diverse human haplotypes will be important to assess population-level variability.

Future development of GRMhor will include incorporation of confidence scoring, integration with raw read mapping for orthogonal validation, and extension to other satellite families and repetitive structures. Application to phased diploid assemblies and epigenetically profiled datasets may further illuminate the role of HOR diversity in centromere function and genome evolution.

Although the 52mer HOR identified in this study is supported by two structurally identical and fully aligned copies in the T2T-CHM13 chromosome 21 assembly, its biological authenticity remains to be confirmed by orthogonal methods. At present, no raw read–level validation has been performed, and the 52mer structure has not yet been resolved in other haplotypes or population assemblies. However, both identified copies are internally consistent, exhibit no divergence across monomer types, and are embedded in canonical alpha satellite arrays.

Importantly, the region containing the 52mer HOR overlaps with the centromeric domain of chromosome 21 in the CHM13 cell line, which was shown to be enriched for CENP-A binding [7]. While that study did not resolve the HOR structure, the functional annotation supports the biological relevance of this locus. Future analyses involving raw long-read alignments, phased haplotypes, and population-scale assemblies will be essential to verify the recurrence, variability, and centromere association of the 52mer HOR across individuals.

In our accompanying study [56], we provide a detailed comparison of GRMhor with other available tools for HOR detection, including NTRprism, HORmon, and HiCAT. Unlike these methods, which are often limited to canonical or Willard-type HORs, GRMhor is capable of detecting complex and highly divergent structures, including cascading HORs and arrays with partial or no canonical units. For instance, HORmon is specifically designed for reference-guided annotation of known centromeric repeats, whereas GRMhor can de novo identify novel HOR configurations from any tandem monomer array. Furthermore, while NTRprism provides fast, signal-based detection, it lacks fine-grained structural output, which GRMhor supplies through detailed alignment schemes and distance maps.

Additionally, we have benchmarked the runtime performance of GRMhor against NTRprism using the full T2T-CHM13 chromosome 20 assembly. GRMhor completed the full analysis in ~35 min (Apple M3 Max, 36 GB RAM), compared to 125 s for NTRprism. While this illustrates that GRMhor is computationally more demanding, the tradeoff is justified by its superior resolution and structural precision. Users are also encouraged to extract and process individual HOR blocks for faster runtime and clearer output.

## 4. Conclusions

In this study, we applied the GRMhor algorithm to the T2T-CHM13 assembly of human chromosome 21 and precisely delineated the structure of alpha satellite higher-order repeats, including a novel 52mer—the longest canonical HOR unit identified in the human genome to date. Our analysis also revealed complex organizational patterns, such as cascading HORs and multiple subfragment repeat types, demonstrating the structural richness of the centromeric region in this chromosome.

These findings highlight the importance of complete telomere-to-telomere assemblies for uncovering genomic architectures that were previously unresolved in reference builds like hg38. By enabling detailed characterization of repeat structures beyond the capabilities of existing tools, GRMhor contributes a valuable approach for advancing our understanding of centromere biology, genome evolution, and repetitive sequence organization.

## 5. Methods

The reference genome sequences T2T-CHM13v2 for chromosome 21 were obtained from NCBI (www.ncbi.nlm.nih.gov/datasets/genome/GCF_009914755.1/) (accessed on 30 June 2025). Our GRMhor workflow integrates two tools: MonFinder and GRMhor, both publicly available at github.com/gluncic/GRM2023. MonFinder is a tool for detecting alpha satellite monomers based on pairwise alignment with a consensus sequence. The resulting monomer list serves as input for GRMhor, which identifies and visualizes HORs through a set of computational steps. For a detailed description of both algorithms and their applications, see Ref. [56]

## Figures and Tables

**Table 1:** Frequency of MD points of different periods. The 11mer HOR exhibits a total of 1404 MD points. Notably, periods 7 and 4 represent subfragments of the 11mer HOR. The magnitude of GRM peaks, i.e., the number of HOR copies, increases with the number of MD points.

No. of MD Points	Period	Repeat Pattern	Number of HOR Copies
1404	11	major Cascading 11mer HOR	182
221	23	Willard’s-type 23mer HOR	13
190	7	subfragment of Cascading 11mer HOR	
178	4	subfragment of Cascading 11mer HOR	
136	25	Cascading 25mer HOR	6
92	9	subfragment of Cascading 11mer HOR	
62	20	subfragment of Cascading 11mer HOR	
52	52	Willard’s-type 52mer HOR	2

## Data Availability

The MonFinder and GRMhor (python applications) are freely available at github.com/gluncic/GRM2023. Reference genome sequences for chromosome 21 T2T CHM13v2 are freely available via the National Center for Biotechnology Information official website https://www.ncbi.nlm.nih.gov/datasets/genome/GCF_009914755.1/ (accessed on 30 June 2025).

## Supplementary Materials

The following supporting information can be downloaded at: https://www.mdpi.com/article/10.3390/genes16080885/s1. Figure. S1. (separate file) Cascading 11mer alpha satellite HOR alignment. Start position 10,963,339 bp and end position 11,305,104 bp in T2T-CHM 21. The number on the left side indicate the initial position of the first monomer in each row of HOR copy; Figure. S2. 23/25mer alpha satellite HOR alignment. Start position 8,180,952 bp and end position 8,253,585 bp in T2T-CHM13. The numbers on the left side indicate the initial position of the first monomer in each HOR copy; Table S1. Canonical 11mer consensus sequence; Table S2. Canonical 23/25mer consensus sequence;
