# Supplementary material for: Precise Identification of Higher-Order Repeats (HORs) in T2T-CHM13 Assembly of Human Chromosome 21—Novel 52mer HOR and Failures of Hg38 Assembly
Source: Genes (Basel). 2025 Jul 27;16(8):885. doi: 10.3390/genes16080885 (PMC12385485; doi:10.3390/genes16080885)
Supplement: Supplementary file 1 [file genes-16-00885-s001.zip › genes-3776073-supplementary.pdf]

Supplementary Materials for

Precise Identification of Higher-Order Repeats (HORs) in T2T-CHM13 Assembly of Human Chromosome 21—Novel 52mer HOR and Failures of Hg38 Assembly

Matko Glunčić, Ines Vlahović, Marija Rosandić, Vladimir Paar

Corresponding author: matko@phy.hr

The Supplementary file includes:

Figs. S1 to S2
Tables S1 to S2

Fig. S1. (separate file) Cascading 11mer alpha satellite HOR alignment. Start position 10,963,339 bp and end position 11,305,104 bp in T2T-CHM 21. The number on the left side indicate the initial position of the first monomer in each row of HOR copy.

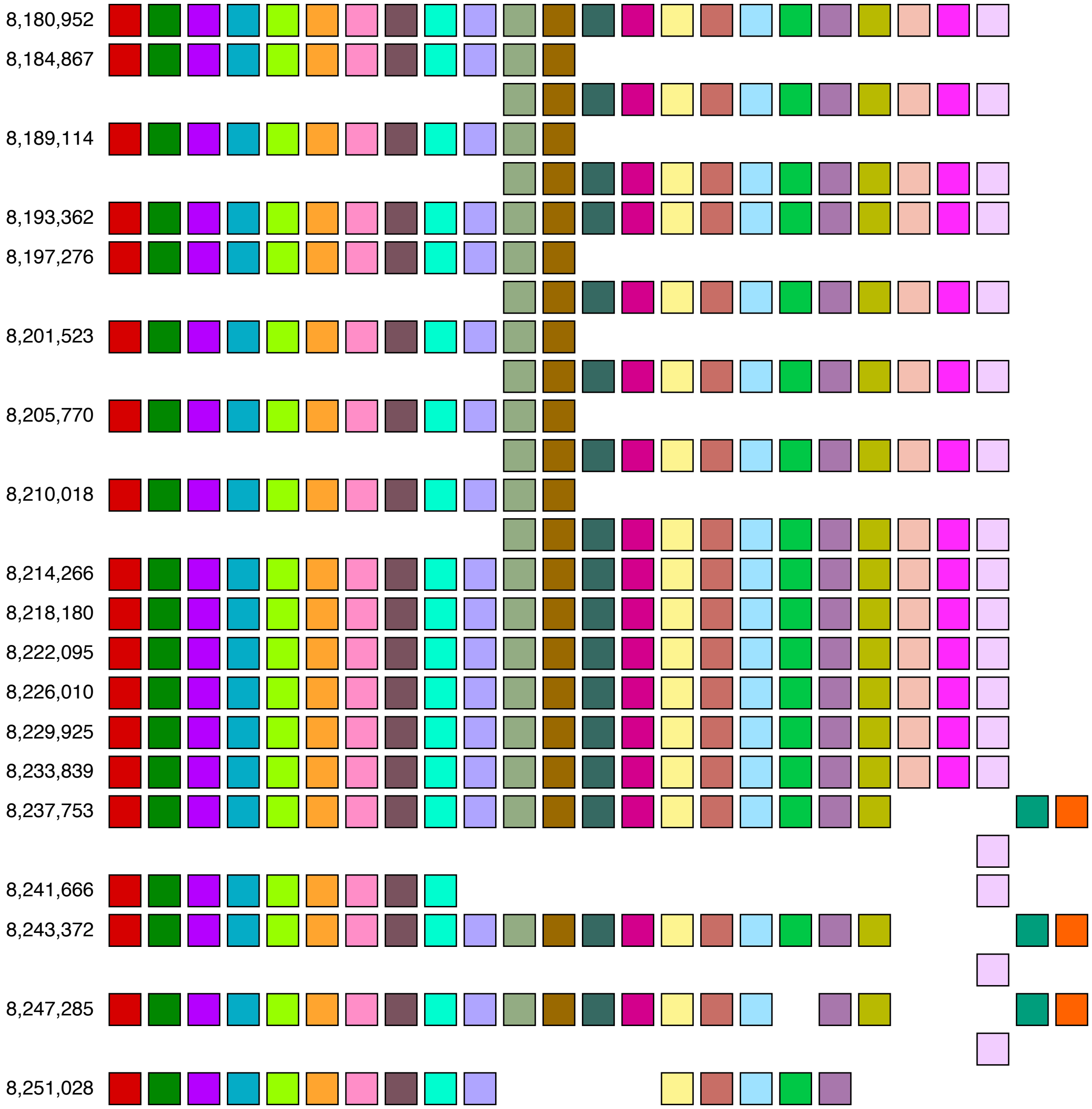

Fig. S2. 23/25mer alpha satellite HOR alignment. Start position 8,180,952 bp and end position 8,253,585 bp in T2T-CHM13. The numbers on the left side indicate the initial position of the first monomer in each HOR copy.

Table S1. Canonical 11mer consensus sequence.

Monomer type t1
TCAGAAACTTGTTCGTGATGTGTGTACTCAACTAAAAGAGTTGAACCTTTCTATTGATAGAGCA
GTTTTGAAACACTCTTTTTGTGGATTCTGCAAGTGGATATTTGGATTGCTTTGAGGATTTCGTT
GGAAGCGGGAATTCGTATAAACACTAGACAGCAGCATTC
Monomer type t2
TTAGAAACTACTTGGTGATATCTGCATTCAAGTCACAGAGTTGAACATTCCCTTACTTTGAGCA
CGTTTGAAACACTCTTTTGGAAGAATCTGGAAGTGGACATTTGGAGCGCTTTGATGCCTTTGGT
GAAAAGGAAACGTCTTCCAATAAAAGCCAGACAGAAGCATTC
Monomer type t3
TCACAAACTTCTTTGTGATGTGTGTCCTCAACTAACAGAGTTGAACCTTTCTTTTGATGCAGCA
ATTTGGAAACACCCTTTTGGTAGAAACTGTAACTGGATATTTGGATAGCTCTAACGATTTCGTT
GGAAACGGGAATATCATCATCTAAAATCTAGACAGAAGCACTA
Monomer type t4
TCAGAAACAAGTTTGTGATGTGTGTACTCAGCTAACAGAGTGGAACCTTTCTTTTTACAGAGCA
GCTTTGAAACTCTATTTTTGTGGATTCTGCAAATTGATATTTAGATTGCTTTAACGATATCGTT
GGAAAAGGGAATATCGTCATACAAAATCTAGACAGAAGCATTC
Monomer type t5
TCAGAAATTTCTTTCTGATGTCTGCATTCAACTCATAGAGTTGAAGATTCCCTTTCATAGAGCA
GGTTTGAAACACTCTTTCTGGAGTATCTGGATGTGGACATTTGGAGCGCTTTGATGCCTACGGT
GAAAAAGTAAATATCTTCCCATAAAAACGAGACAGAAGGATTC
Monomer type t6
TCAGAAACTTGTTTGTGATGTGTGTACCCAGCCAAAGGAGTTGAACATTTCTATTGATAGAGCA
GTTTTGAAACACTCTTTTTGTGGAAAATGCAGGTGGATATTTGGATAGCTTGGAGGATTTCGTT
GGAAGCGGGAATTCAAATAAAAGGTAGACAGCAGCATTC
Monomer type t7
TCAGAAACTACTTTGTGATATCTGCATTCAAGTCACAGAGTTGAACATTCGCTTTCTTAGAGCA
CGTTTGAAACACTCTTTTTGTAGTGTCTGGAAGTGGACATTTGGAGCGCTTTGATGCCTTTGGT
GAAAAAGGGAACGTCTTCCCATAAAAACTAGACAGAAGCATTC
Monomer type t8
TGAGAAACAAGTTTGTGATGTGTGTACTCAGCTAACAGAGTGGAACCTCTCTTTTGATGCAGCA
GTTTGGAAACACTCTTTTTGTAGAAACTGTAAGTGGATATTTGGATAGCTCTAATGATTTCGTT
GGAAACGGGAATATCATCATCTAAAATCTAGACAGAAGCCCTC
Monomer type t9
TCAGAAACTTGTTGGTGATATGTGTCCTCAACTAACAGAGTTGAACTTTGCCATTGATAGAGAG
CAGTTTTGAAACACTCTTTTTGTGGAATCTGCAAGTGGATATTTGGATAGCTTGGAGGATTTCG
TTGGAAGCGGGAATTCAAATAAAAGGTAGACAGCAGCATTC
Monomer type t10
CCAGAAATTTCTTTCGGATATTTCCATTCAACTCATAGAGATGAACATGGCCTTTCATAGAGCA
GGTTTGAAACACTCTTTTTGTAGTTTGTGGAAGTGGACATTTCGATCGCCTTGACGCCTACGGT
GAAAAAGGAAATATCTTCCCATAAAAAATAGACAGAAGCATTC

Table S2. Canonical 23/25mer consensus sequence.

Monomer type t1
TGAGAAACTTCTTTGTGATATTTGCTTTTATCTCATAGAGTTGAAACTTTCTTTTTATTGAGCA
GTTTGGGAACAGTCTTTTTGTAGTATCTGCAAATGGATATTACCAGTGCTTTGAGGCCTATGGT
GAAAAAGGAAATATCTTCACATAAAAACAAGGCGGAAGCATTC
Monomer type t2
TGAGAAACTTCTCTGTGATGGATGCATTCATTTCACAGAGTTAAACCTTTCCTGTGATTGAGCG
GTTTGGAAACAGTAGTTTTTACAATCTGCAGAAGGATACTTGTGAGCCGATTGAGGTCTATGG
GGTGATAAGAAATATGTTCACATAAAAACTAGATAGAAAGTTTC
Monomer type t3
TAGAAACTTCTTTGTGATGTGTGCATTCATCTCACCGACTAGAACCTTTCTTTTGATTGAGCAG
TTTTGAAACACTCTTTTAGCGGAATCTGCAAGTGTTTATTTGGAGCGCATGAGGAATATGGTGG
AAAAGGAATCTTCTTCACATGAAAACGAGACGGAAGCATTC
Monomer type t4
TCAGAAACGTCTTGGTGATGTGTGCATTCATCTCACTGAGTTGAACTTTATTTTGATTGAGCAG
TTTGGAAACAGTCTTTTCTAGTATCTGCAAATGGATATTTTAAGCACTCTGAGGCCTACGGTGA
AAAAGGAAATATCTTCAATATAAATCAGACAGAAGCATTCA
Monomer type t5
TGAGAAACTTCCTTTGAATGGGCGCATTCATCTCACACTGTTGAACTTTTTTTTTGATTGAGCA
CCTTCTAAACAGTCATTTTGTAGAATATGCAAAGGAATATTTGTGAGCCCATTGATGCCTCTGG
GGAAACAGGAAATATCTTCACATAAAAACGAGACAGAATCTTTC
Monomer type t6
TGGAGAAATTTATTTTGATGTGTGCATTCATCTCACACAGTTGAAATTTTCTTTTGATTGAGCA
GTGTGGATACACTCGTTTTGTAGAGTCTGCAAGTGGATATTTGGAGCACTTTGTGGCCTATAGT
GAAAAAGGAAATATCTTCACATAAAAACTAGATAGAAGAATTC
Monomer type t7
TGAGAAACTATTTTGTCATGTGTGACTTCTACTCACCGGGTTGAAACTTTCTGTTGATTGAGCA
GTTTGGAAACAGTCTTTTTGTAGAATCTGCAAATTGATATTTGGAGTGCTTTTGGCCTACGTTG
AAAAACGAAATATCTTCCCATAAAAAGTAGGCAGAAGTTT
Monomer type t8
TGAGAAACTTATTTGTGATCTGTGCATTCATCTCACAGAGTTGAACCTTTCTTTTGATTCAGCA
GTTTTGAAACTGTCGTTTTGTAGAATCTGCAAAGGAATATTTGTGAGCCCATTGAGGCTTCTGG
GGTGATAGGAAATATCTTCACATAAAAACTAGACAGATACTTTC
Monomer type t9
TGAGAAACTTCTTTGTGACGTGTGCATTCAACTCATGGAGTTCAACCTTTCTTTTGATTCAGCA
GTTTGGAAACAGTCTTTTTACAGTATCTGCAAATGGCTATTTGGAGAGCTTTGAGGCCTATGGT
GGAAAAGGAAATCTCTTCCCATAAAAACTAGACAGCAGCATTC
Monomer type t10
TGAGAAACTTCTTTGTGATGTGTGCATTCATCTCACAGAGTTGAATCTTTCTTTTGTTTGAGCA
GTTTTGAAACTCTCTTTCTGTAGAATCTTCAAGTGGATATTTTCAGCGCTTTGAGGCCTATGGT
GGAAAAGAAAATATCTTCACATAAAAACTAGTCAGAAGCATTC
Monomer type t11
TGAGGAACTTCATGATGTGTGCATTCATCTCAAAGAGTTGAACTTTTCTTTTGATTGAGCAGCT
TTGAAAAACTCTTTCTGCAGAATCTGCAAGTTGATATTTGGAGTGCTTTGTGGCCTATAGTAGA
AAAGGAAATATCTTTACATAAAACTAGACAGAAGCATTC
Monomer type t12

TGAGAAACTTCTTAATGATGTGTGCATTCATCTCACAGAGTTGAAACTTTCTTTTGATTGAGCC
GTTTGGAAACACTCTTTTAGTAGAAACTGCAAGGGGATATTTGGAGCGTTTTGTGGTCTATGGT
AGAAAAGGATATATCTTCACATAAAAATAGAAGCATTC

Monomer type t13

TAAGTAATCTCTTTGAGATGCGTGCATTCAACTCACAGAGTTGGACATTTCCTTTGATTGAGCA
GTGTGGAAACAGTCTTTTTGCAGTATCTGCAAACGGATATTTGCAGCACTTTCAGGCCTATAGT
AGGAAAGGAAATATCTTCACATAAAAACTAGACAGAAAATTAC

Monomer type t14

TAAGAAAGTGCTTTGTGACGTGTGCATTCATCTCACAGTGTTGAACCTTTCTTTTGATTGAGCA
GTTTTGAAACACTCTTATTGTAGAATCTGCAAGTGGATATTTGGAGAGTTTGAGGCCACTGGTG
GAAAAGCAAATATCTTCACATCAAAACTAGACAGAATCATTA

Monomer type t15

TGAGAAACTGCTTTGTGATGCGTGCATTCATCACCAGAGTTGAGTTTCTCTTTTGATTGAACAG
TTTTGAAACACTCTTTCTGTAGAATCTGAAAGGGATATTTGGAGCGCTTTGCAGCCTATGGTGA
AAAAGGAAATATCTTCACATAAAAGCTAGACAGAAGCATTC

Monomer type t16

TGAGAAACTTCTTTGTGATGTGCTCATTCAACTCACAGAGTTGAACTTTTCTTTTGTTTGAGCA
GTTTGCAAACAGTCTTTTTGTAGAATCTGCAAGTGGATATTAGGAGTGCATTACGGCCTATAGT
GGAGAATGAAATATCTTCACATAAAAACTAGACAGAAACATTA

Monomer type t17

TGAGAAACTTCTTTGTGATGTGCTCATTCAACTCACAGAGTTGAGCTTTTCTTTTGATTGAGCA
GTTTGGAAACAGTCTTTTTGTAGAATCTGCAAGTGGATATTTGGAGCGCATGACGGCCTATAGT
GGAAAAGGAAATATATTCACATAAAAACTAGACAGAAGCATTC

Monomer type t18

TGAGAAGCTTCTTTGTGATGTGTGCGTCCATCTCGAAGAGTTGAACCTTTCTTTTGATTGAGCA
TTTTTGAAGCACTCTTTTTGTAGAATCTTCAAGTGGATATTTGGAGGGTTTGTGGCCTGTGGTG
GAAAAGGAAATATATTCACATAAAAACTAGATAGAAGCATTC

Monomer type t19

TGAGAAAGTTCTCTGTGTTGTATGCAGTCATATCTCAGACATGAAACTTTCTTTGGTACAGCAG
TTTTAAAACACTCTTTTTGGAGATTCTGAAAGTAGATATTTGGAGAGACTTGAGGACTACGGTG
GAAAAGGAAATATCTTCACAAAAAAACTAGACAGAAACATTC

Monomer type t20

TTAGAAACTCCTTTGTGATGTGTGCATTCATCTCACAGACTTCAAACTTTCTATTGATTGAGCA
GTTTTGAAACACTCTTTTTGTAGAATCTGCCAGTGGATATTTGGAGCGCTCTGTGGCCCATAGT
GGAAAAGGAAATATCTTCATAAGAAAAATAAACAGAAGCACTT

Monomer type t21

TGAGAAACACCCTTGTGATGTGTGCATTCATCATGCACAGTTGAACTTTCTTTTGATTGAGCAG
TTTGGATACAGTCATTTGTATTATCTGTAAATGGATATTTGGAGTGTACTGAGGCCTATGGTGA
AAAAGGAAATATCCTCACATAAAATTCAGATGGAAGCATTC

Monomer type t22

TGAGAAACTTCCTAGGAAGGTGTATTTTCGTCTCACACTGTTAAACCCGTCTTTTGATTGAGCA
GCTTCGATACAGTCATTTAGTAGAATATGAAAGGGAATATTTGAGAGCCCATTGAGGCCTCTGG
GGAAATAAGAAATATCTTCACCTAAAAACAAGACAAAATCTTTC

Monomer type t23

TGAGAAACTTCTTTTTGATGTCTGCATTCATCTCACAGAGTTGAACCTTTCTTTTGATTGAGCA
GTTTTGAAACGCTCTATTTGTAGTATCTGCAAGTGGATATTTGGAACGCTTTGAGGCCTATAGT
GGAAAAGGAAATATCTTCACATAAAAAACTAGAAAGAAGAATTC
